# YAP1 Knockdown Reduces IL-1β-Induced Human Chondrocyte Inflammation and Promotes Human MSC Chondrogenesis

**DOI:** 10.3390/ph19060859

**Published:** 2026-05-29

**Authors:** Liru Wen, Sibylle Grad, Laura B. Creemers, Martin J. Stoddart

**Affiliations:** 1 AO Research Institute Davos, 7270 Davos Platz, Switzerland; wenliru2018@email.szu.edu.cn (L.W.); sibylle.grad@aofoundation.org (S.G.); 2 Department of Orthopaedics, University Medical Center Utrecht, 3584 CX Utrecht, The Netherlands; laura.creemers@maastrichtuniversity.nl; 3 Faculty of Health, Medicine and Life Sciences, Maastricht University, 6229 ER Maastricht, The Netherlands

**Keywords:** YAP1, chondrocytes, IL-1β, MSC, TGF-β1, inflammation, chondrogenesis, hypertrophy

## Abstract

**Background**: Yes-associated protein 1 (YAP1), a key effector of the Hippo signaling pathway and mechanosensitive transcriptional coactivator, plays a complex role in osteoarthritis (OA) and cartilage regeneration. While YAP1 is essential for tissue homeostasis, its dysregulation has been implicated in both inflammatory and degenerative joint pathologies. However, its precise function remains ambiguous. **Methods**: We silenced YAP1 with small interfering RNA (siYAP1) in two human-cell-based models relevant to OA pathogenesis and cartilage repair: (1) IL-1β (10 ng/mL)-stimulated articular chondrocytes in monolayer and pellet cultures, and (2) TGF-β1 (10 ng/mL)-induced chondrogenesis in MSC pellet cultures. Outcome measures comprised YAP1 nuclear localization; inflammatory/catabolic markers in chondrocytes (IL6, IL8, ADAMTS5, MMP13); and, in MSC pellets, chondrogenic or hypertrophic markers (COL2A1, ACAN, RUNX2, MMP13, COL10A1) together with glycosaminoglycan (GAG) deposition. Statistical significance was assessed using an ANOVA or Friedman test with post hoc correction (Tukey or Dunn’s test, respectively); *p* < 0.05 was considered significant. **Results**: In human chondrocytes, siYAP1 reduced IL-1β-induced nuclear YAP1 localization and suppressed pro-inflammatory mediators IL6 and IL8, indicating an anti-inflammatory effect. YAP1 silencing also downregulated ADAMTS5 expression in 2D monolayers but not in 3D pellet cultures, suggesting reduced regulatory influence in the three-dimensional environment. Notably, MMP13 expression was paradoxically increased following YAP1 knockdown, underscoring the complexity of YAP1’s role in catabolic regulation. In MSC chondrogenesis, siYAP1 enhanced TGF-β1-induced chondrogenesis by increasing COL2A1 and ACAN expression and promoting GAG deposition on day 21. Additionally, it reduced hypertrophic markers RUNX2 and MMP13 on day 7, though COL10A1 remained elevated compared to negative siRNA, indicating only partial suppression of hypertrophic differentiation. Nuclear YAP1 levels were increased by day 21 despite reduced mRNA, suggesting post-transcriptional regulation or enhanced nuclear translocation. **Conclusions**: These findings demonstrate that YAP1 knockdown exerts context-specific anti-inflammatory and pro-chondrogenic effects while partially mitigating hypertrophy. However, divergent outcomes, namely elevated MMP13 in chondrocytes and upregulated COL10A1 in MSCs, indicate that YAP1 silencing does not uniformly suppress inflammation or hypertrophy. YAP1 represents a potential therapeutic target for OA, but its modulation requires careful consideration of cellular context, siRNA delivery method, and timing to optimize outcomes for cartilage repair and joint preservation.

## 1. Introduction

Osteoarthritis (OA) is a highly prevalent degenerative joint disease characterized by progressive cartilage degradation, synovial inflammation, and subchondral bone remodeling, and remains a major cause of disability globally [1]. A major driver of OA pathogenesis is the dysregulation of chondrocyte homeostasis by pro-inflammatory cytokines, particularly interleukin-1β (IL-1β), which induces catabolic processes and inhibits extracellular matrix synthesis [2]. Under such inflammatory conditions, chondrocytes undergo phenotypic shifts toward senescence or apoptosis, further compromising cartilage integrity [3]. Mesenchymal stem cells (MSCs) are promising candidates for cartilage regeneration, given their capacity for chondrogenic differentiation and immunomodulation, thereby facilitating cartilage repair and mitigating the inflammatory microenvironment [4]; however, their therapeutic efficacy is constrained by their predisposition toward hypertrophic differentiation [5].

Yes-associated protein 1 (YAP1), a key transcriptional coactivator, plays a pivotal role in mechano-transduction [6], inflammation [7], and stem cell fate decisions [8]. Its nuclear translocation is constrained by the Hippo pathway, whose activation retains YAP1 in the cytoplasm [9]. Once in the nucleus, YAP1 interacts with TEAD transcription factors to regulate gene expressions and therefore can influence multiple processes simultaneously [10].

Previous studies have reported conflicting roles for YAP1 in OA; one demonstrated that YAP1 activation attenuates IL-1β-induced inflammation in bovine chondrocytes [11], whereas another showed that YAP1 knockdown inhibits IL-1β-induced catabolic gene expression in murine chondrocytes [12]. However, its role in human chondrocytes remains unclear, as most prior studies have employed bovine or murine systems. Additionally, YAP1 knockdown promotes chondrogenesis in human synovial MSCs at day 7 [13], while its overexpression inhibits early chondrogenesis in both murine and human models [14,15]. Inducing MSC chondrogenesis is an important strategy for cartilage repair [16]; however, YAP1’s long-term role in MSC chondrogenesis remains unknown. These knowledge gaps limit the translational relevance of YAP1-targeting strategies for OA treatment.

While pharmacological inhibitors of YAP1 demonstrate therapeutic effects in animal tumor models [17], they may lack specificity and could induce drug resistance. RNA interference (RNAi) technologies, particularly small interfering RNA (siRNA), offer targeted, stable, and sustained suppression of YAP1 expression, potentially overcoming limitations of pharmacological approaches [18]. Despite advancements in siRNA delivery application in monolayer cultures, the long-term effects of YAP1 silencing in three-dimensional (3D) pellet models, which more closely mimic in vivo conditions, remain underexplored.

In this study, we investigated the effects of YAP1-targeting siRNA (siYAP1) in two complementary human cell models: (1) IL-1β-induced inflammation in articular chondrocytes cultured in monolayer and pellets, and (2) Transforming Growth Factor Beta 1 (TGF-β1)-driven chondrogenesis in MSC pellet cultures. Monolayer cultures enable efficient analysis of inflammatory signaling and proliferation, while 3D pellet cultures better preserve the chondrogenic phenotype and cell–matrix interactions, thereby providing complementary insights into OA-related processes [19]. Our findings demonstrate that siYAP1 attenuates IL-1β-induced inflammation in chondrocytes, enhances chondrogenesis and partially inhibits hypertrophic differentiation in MSCs, supporting YAP1 as a potential therapeutic target for osteoarthritis treatment and cartilage regeneration. Preliminary results were presented at the 2024 European Orthopaedic Research Society (EORS) Annual Meeting in Aalborg, Denmark [20].

## 2. Results

### 2.1. YAP1 siRNA Suppresses IL-1β-Induced Nuclear Localization of YAP1 and Proliferation in Monolayer Chondrocytes

**Figure 1:**
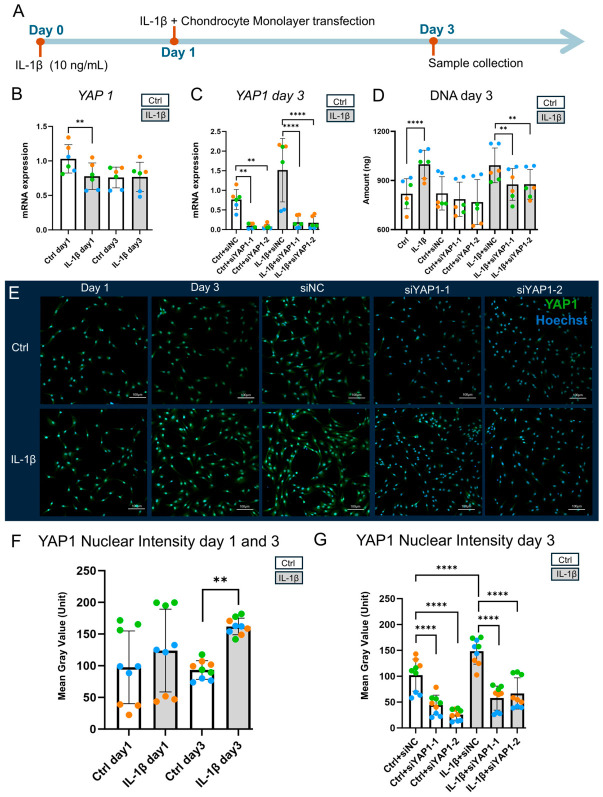
Effects of IL-1β on YAP1 nuclear localization in chondrocytes and the modulatory role of YAP1-targeting siRNA in monolayer culture. (**A**) Chondrocytes were treated with IL-1β (10 ng/mL) from day 0 onward and transfected with YAP1 siRNA (siYAP1-1 or siYAP1-2) using Lipofectamine™ RNAiMAX on day 1 (*n* = 3, duplicates). (**B**) YAP1 mRNA expression following IL-1β treatment at days 1 and 3. (**C**) YAP1 mRNA expression on day 3 following siRNA-mediated knockdown. (**D**) DNA content analysis after YAP1 knockdown on day 3. (**E**) Immunofluorescence staining of YAP1 on day 3. Scale bar: 100 μm. (**F**,**G**) Quantification of nuclear YAP1 localization. ** *p* < 0.01, **** *p* < 0.0001. Different colors indicate individual donors. Ctrl: control; siNC: negative-control siRNA; siYAP1: YAP1-targeting siRNA.

We investigated the role of IL-1β in regulating YAP1 nuclear localization in chondrocytes and assessed the therapeutic potential of YAP1-targeting siRNA in a monolayer culture. Chondrocytes were treated with IL-1β (10 ng/mL) on day 0 (Figure 1A) and transfected with siRNA using Lipofectamine™ RNAiMAX on day 1. IL-1β treatment reduced YAP1 mRNA expression on day 1 but had no effect by day 3 (Figure 1B). Transfection with YAP1-specific siRNAs (siYAP1-1 and siYAP1-2) effectively suppressed YAP1 mRNA levels in both untreated and IL-1β-treated cells (Figure 1C). IL-1β resulted in increased DNA content, suggesting enhanced cell proliferation (Figure 1D), while siYAP1-1 and siYAP1-2 attenuated this proliferative effect compared to negative-control siRNA (siNC) in the IL-1β-treated cells. Importantly, siYAP1 did not reduce DNA content in the absence of IL-1β, ruling out non-specific cytotoxicity. Despite reduced YAP1 mRNA levels at day 1, immunocytochemistry showed a trend toward increased nuclear YAP1 localization in the IL-1β group at day 1 and a significant increase at day 3 compared to controls (Figure 1E,F), suggesting possible post-transcriptional regulation. Notably, compared to the siNC-treated cells, nuclear YAP1 protein levels decreased in the siYAP1-1- and siYAP1-2-treated cells (Figure 1E,G).

### 2.2. YAP1 siRNA Partially Alleviates IL-1β-Induced Inflammation in Monolayer Chondrocyte Culture

**Figure 2:**
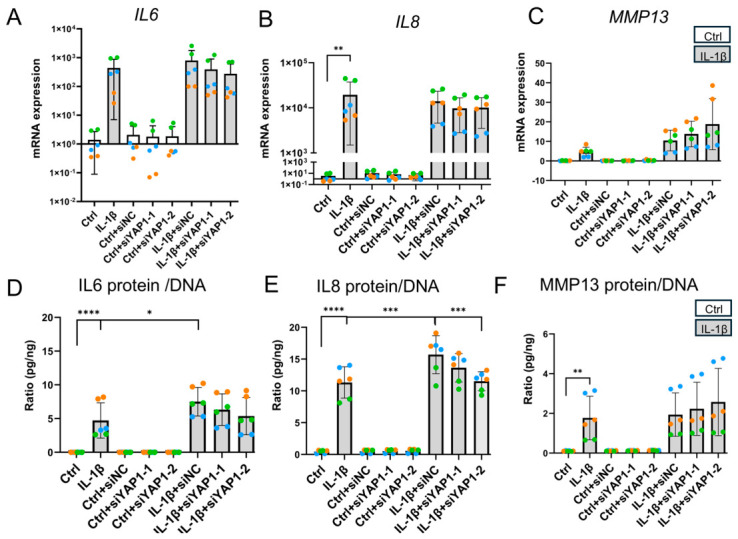
siYAP1 partially attenuates IL-1β-induced inflammatory and catabolic responses in chondrocytes. (**A**–**C**) mRNA expression of IL6, IL8 and MMP13 across treatment groups (*n* = 3, duplicates). (**D**–**F**) IL6, IL8, and MMP13 levels normalized to DNA content. * *p* < 0.05, ** *p* < 0.01, *** *p* < 0.001, **** *p* < 0.0001. Different colors indicate individual donors. From A, the *Y*-axis is displayed on a log10 scale due to high data variability; error bars extending below zero are not visualized on this scale. Ctrl: control; siNC: negative control siRNA; siYAP1: YAP1-targeting siRNA.

As shown in Figure 2A–C, IL-1β led to a significant upregulation of IL8, and a non-significant upregulation of IL6 and MMP13 mRNA expression compared to the controls, suggesting activation of inflammatory and catabolic pathways. IL-1β + YAP1 siRNAs (siYAP1-1 and siYAP1-2) reduced IL6, IL8, and ADAMTS5 mRNA expression while increasing MMP13 mRNA relative to IL-1β + siNC; however, none of these differences reached statistical significance (Figure 2A–C for IL6, IL8, MMP13; Figure S1A for ADAMTS5). 

Since siYAP1 also reduced DNA content, cytokine levels were then normalized to DNA. After normalization, IL6 protein/DNA and IL8 protein/DNA were lower in IL-1β-treated siYAP1 cells than in IL-1β-treated siNC cells, while MMP13/DNA showed no difference (Figure 2D–F). ADAMTS5 protein levels were assessed by ELISA but were undetectable in all groups, likely due to low protein abundance, as suggested by high Cq values.

Notably, compared to IL-1β treatment alone in monolayer culture, IL-1β + siNC further increased IL-6 and IL-8 protein/DNA ratios significantly (Figure 2D,E).

### 2.3. Sustained YAP1 Suppression by siYAP1-2 in Chondrocyte Pellet Culture Under IL-1β Stimulation

**Figure 3:**
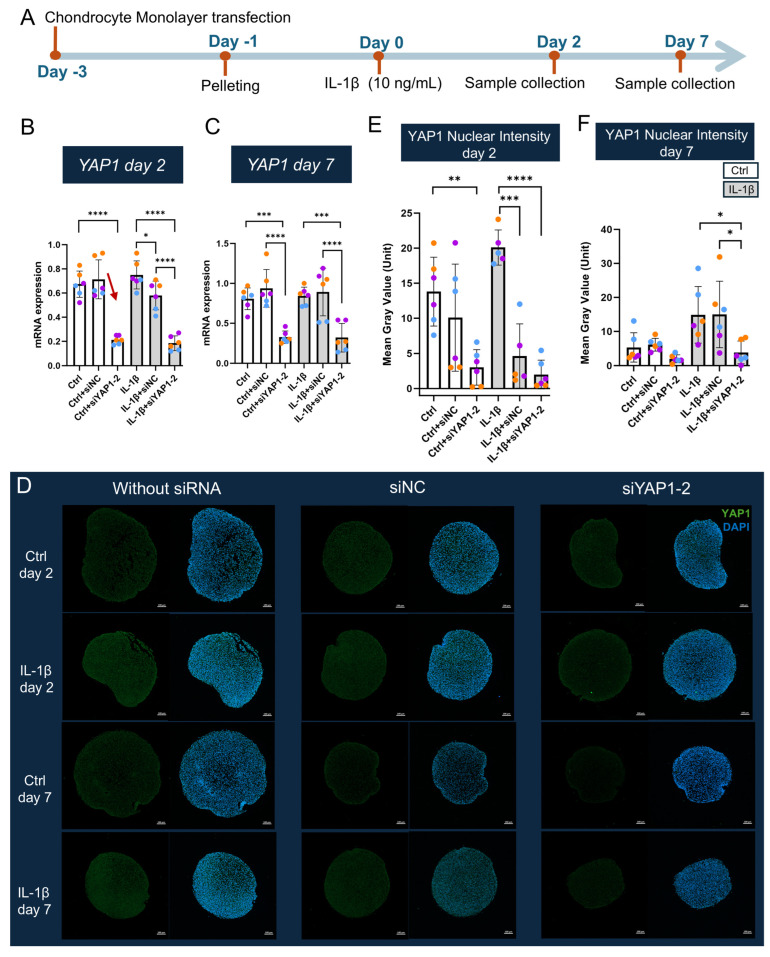
Effects of IL-1β on YAP1 nuclear localization and the modulatory role of YAP1-targeting siRNA in chondrocyte pellet culture. (**A**) Chondrocytes were transfected with siYAP1-2 on day −3 and treated with IL-1β (10 ng/mL) from day 0 onward. (**B**,**C**) YAP1 mRNA expression on days 2 and day 7 following IL-1β treatment and siRNA transfection (*n* = 3, duplicates). (**D**) Immunofluorescence staining of YAP1. Scale bar: 100 μm. (**E**,**F**) Quantification of nuclear YAP1 localization. * *p* < 0.05, ** *p* < 0.01, *** *p* < 0.001, **** *p* < 0.0001. Different colors indicate individual donors. Ctrl: control; siNC: negative-control siRNA; siYAP1: YAP1-targeting siRNA.

We evaluated the silencing durability of siYAP1-2, selected for its more effective suppression of IL-1β-induced IL6/DNA and IL8/DNA ratios compared to siYAP1-1 in chondrocyte monolayer culture. Chondrocytes were transfected with siYAP1-2 in monolayer culture on day −3. On day −1, the cells were formed into pellets, and IL-1β (10 ng/mL) was added from day 0 onward (Figure 3A). IL-1β + siYAP1-2 significantly reduced YAP1 mRNA levels on day 2 and maintained suppression through day 7 compared to IL-1β + siNC (Figure 3B,C), with no impact on DNA content at either timepoint (Figure S1A,B). Nuclear YAP1 localization, assessed via immunofluorescence (Figure 3D), was reduced in siYAP1-2-treated cells compared to siNC, showing a non-significant trend at day 2 and a significant reduction at day 7, respectively (Figure 3E,F). These results confirm siYAP1-2 achieves durable YAP1 suppression in chondrocyte pellet culture. IL-1β exhibited a non-significant trend toward increased nuclear YAP1 on day 2 and day 7 compared to the Ctrl (Figure 3E,F). IL-1β + siNC-treated cells transiently reduced nuclear YAP1 on day 2 versus IL-1β alone, but this effect dissipated by day 7 (Figure 3E,F).

### 2.4. siYAP1-2 Partially Alleviates IL-1β-Induced Inflammation in Chondrocyte Pellets

**Figure 4:**
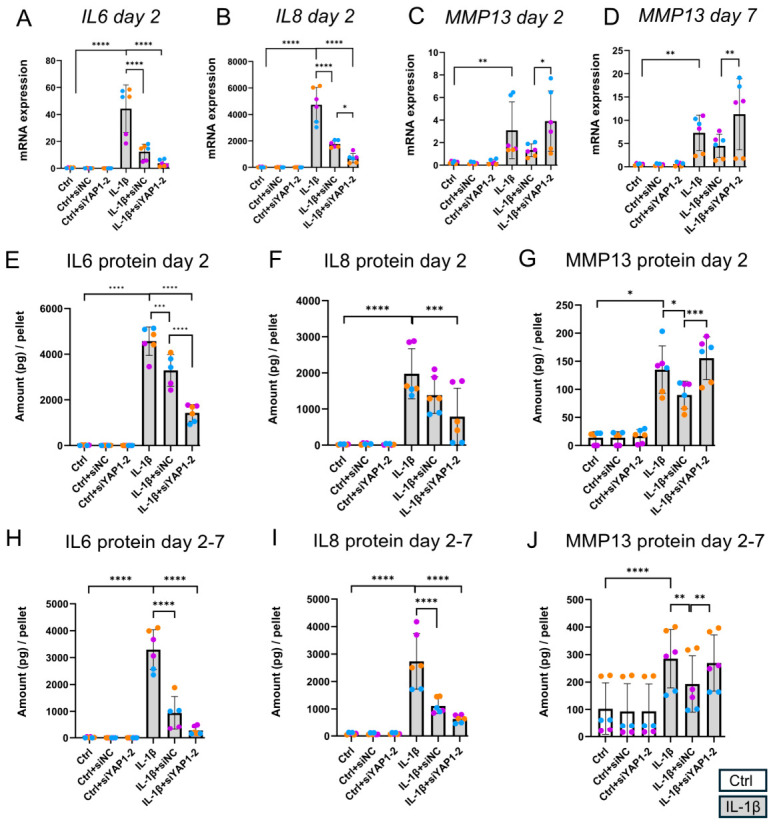
siYAP1-2 partially attenuates IL-1β-induced inflammatory responses in chondrocyte pellets. (**A**–**C**) mRNA expression of IL6, IL8 and MMP13 at day 2 (*n* = 3, duplicates). (**D**) mRNA expression of MMP13 at day 7. (**E**–**G**) Secretion of IL6, IL8, and MMP13 per pellet on day 2. (**H**–**J**) Secretion of IL6, IL8, and MMP13 per pellet from day 2 to day 7. * *p* < 0.05, ** *p* < 0.01, *** *p* < 0.001, **** *p* < 0.0001. Different colors indicate individual donors. Ctrl: control; siNC: negative-control siRNA; siYAP1: YAP1-targeting siRNA.

The effects of YAP1 knockdown on IL-1β-induced inflammation were evaluated in chondrocyte pellet cultures at both transcriptional and protein levels on days 2 and 7. As shown in Figure 4A–C, IL-1β significantly upregulated IL6, IL8, and MMP13 mRNA expression on day 2 relative to the Ctrl. Notably, IL-1β + siYAP1-2 reduced IL6 and IL8 mRNA but increased MMP13 mRNA compared to IL-1β + siNC on day 2 (Figure 4A–C). By day 7, IL-1β + siYAP1-2 had no significant effect on IL6 or IL8 RNA levels compared to IL-1β + siNC (Figure S1F,G), though it continued to elevate MMP13 expression (Figure 4D). No difference in ADAMTS5 mRNA expression was detected by day 7 (Figure S1E).

At the protein level, ADAMTS5 was undetectable in all groups by ELISA, likely due to low mRNA abundance. ELISA confirmed that YAP1 knockdown reduced IL6 and IL8 secretion at day 2, while increasing MMP13 levels (Figure 4E–G). From day 2 to day 7, IL8 remained suppressed and MMP13 levels increased. IL6 secretion decreased in 2 out of 3 donors during this period (Figure 4H–J). These findings suggest that YAP1 knockdown partially mitigates IL-1β-induced inflammation in pellet culture but also enhances MMP13 expression.

Interestingly, IL-1β + siNC reduced IL6, IL8, and MMP13 expression compared to IL-1β alone on day 2, suggesting that transfection may influence the inflammatory response in pellet culture (Figure 4A–C). Similarly, IL-1β + siNC showed a non-significant trend toward reduced inflammatory gene expression of IL6, IL8 and MMP13 relative to IL-1β alone on day 7 (MMP13: Figure 4D; IL6, IL8: Figure S1F,G). siNC also reduced IL-1β-induced secretion of IL6, IL8, and MMP13 on days 2 and 7 compared to IL-1β alone at day 2 and day 7 (Figure 4E–J), further supporting an effect of transfection on the inflammatory profile.

To exclude potential effects of siNC, we compared IL-1β + Mock (Lipofectamine™ RNAiMAX only) and IL-1β + siNC groups. Analyses included IL6, IL8, ADAMTS5, and MMP13 mRNA, as well as IL6 and IL8 protein/DNA in monolayer cultures (Figure S1L–Q), and YAP1, IL6, IL8, and MMP13 mRNA and protein, together with YAP1 nuclear intensity in pellets at day 2 and 7 (Figures S1R,S and S2A–N). The only difference observed was a reduction of IL8 mRNA in the IL-1β + siNC group compared with IL-1β + Mock at day 2 in pellets (Figure S2E). Importantly, YAP1 knockdown caused a further reduction in IL8 mRNA compared with siNC, supporting the conclusion that YAP1 silencing downregulates IL8 at day 2 (Figure 4B). No other differences were detected.

### 2.5. siYAP1-2 Suppresses TGF-β1-Induced YAP1 Expression and Nuclear Localization in MSC Pellets

**Figure 5:**
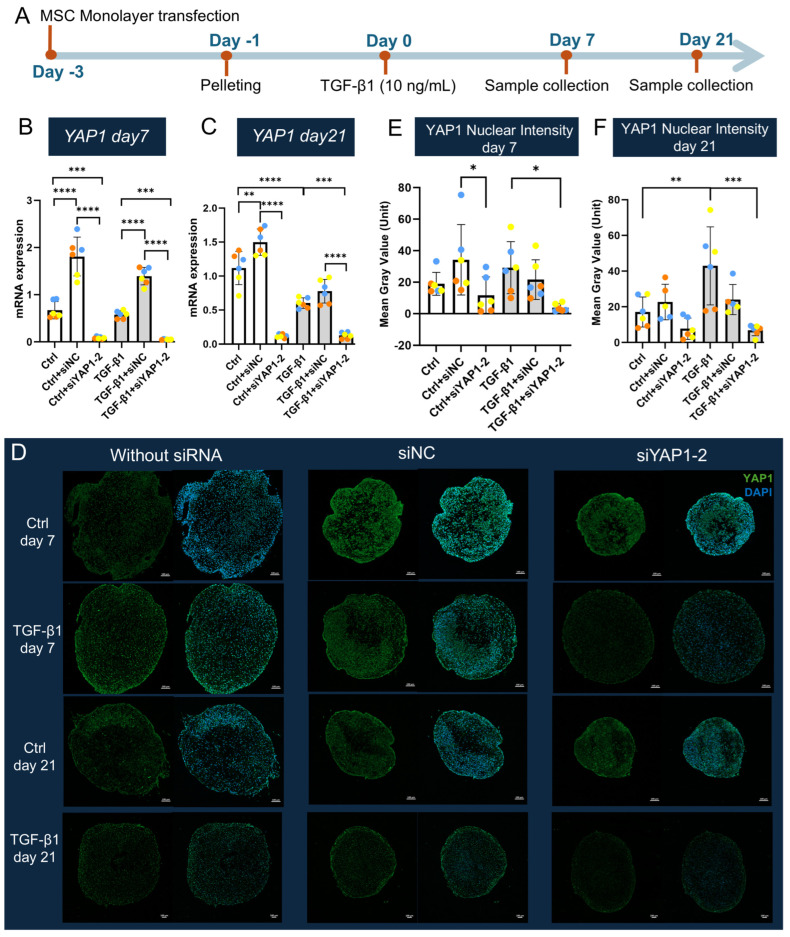
Effects of TGF-β1 on YAP1 nuclear localization and the modulatory role of YAP1-targeting siRNA in MSC pellet culture. (**A**) Experimental timeline: MSCs were transfected with siYAP1-2 on day-3 and treated with TGF-β1 (10 ng/mL) from day 0 onward. (**B**,**C**) YAP1 mRNA expression on days 7 and 21 following TGF-β1 stimulation and siRNA transfection. (**D**) Immunofluorescence staining of YAP1. Scale bar: 100 μm. (**E**,**F**) Quantification of nuclear YAP1 localization. Different colors indicate individual donors. Ctrl: control; siNC: negative-control siRNA; siYAP1: YAP1-targeting siRNA. * *p* < 0.05, ** *p* < 0.01, *** *p* < 0.001, **** *p* < 0.0001.

We investigated the effects of YAP1 knockdown on MSC pellet cultures using siYAP1-2. MSCs were transfected in monolayer with siYAP1-2 on day -3, followed by TGF-β1 treatment (10 ng/mL) from day 0 onward (Figure 5A). siNC treatment increased YAP1 mRNA expression in control pellets at both time points, and in TGF-β1-treated pellets at day 7 but not at day 21 (Figure 5B,C). YAP1 mRNA levels were significantly reduced in siYAP1-2-treated pellets on both days 7 and 21 compared to the siNC group (Figure 5B,C). Notably, TGF-β1 reduced YAP1 mRNA levels at day 21 compared to the control.

DNA content analysis showed no significant difference between siYAP1-2 and siNC in TGF-β1-treated cells at either time point (Figure S1C,D). Immunofluorescence staining demonstrated a reduction in nuclear YAP1 intensity in TGF-β1 + siYAP1-2-treated MSCs at both days 7 and 21 compared to TGF-β1 + siNC, although the differences were not statistically significant (Figure 5D–F). These findings indicate that siYAP1-2 effectively and durably suppresses TGF-β1-induced YAP1 expression and reduces nuclear localization in MSC pellets. Interestingly, TGF-β1 increased nuclear YAP1 localization at day 21 compared to the control, despite decreased mRNA expression, suggesting potential post-transcriptional regulation.

### 2.6. YAP1 Knockdown Enhances GAG Production and Chondrogenic Marker Expression During MSC Chondrogenesis

**Figure 6:**
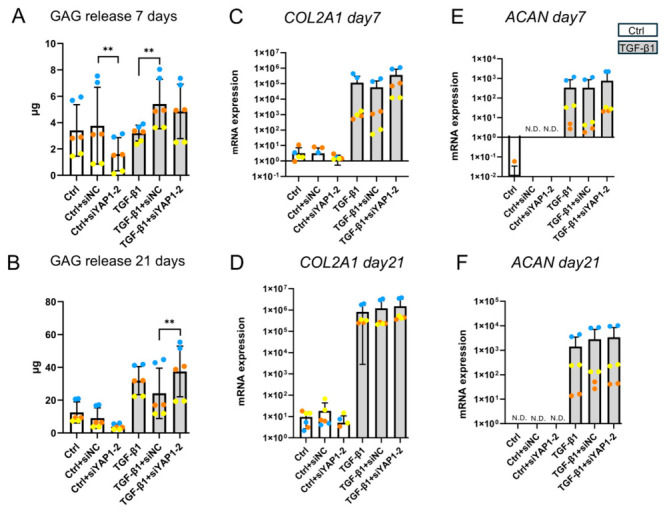
Impact of YAP1-targeting siRNA on chondrogenesis in MSC Pellets. (**A**,**B**) GAG releases per pellet on days 7 and 21. (**C**,**D**) COL2A1 mRNA expression at days 7 and 21 with or without YAP1 knockdown. (**E**,**F**) ACAN mRNA expression at days 7 and 21 with or without YAP1 knockdown. From (**C**–**F**), the Y-axis is displayed on a log10 scale due to high data variability; error bars extending below zero are not visualized on this scale. Different colors indicate individual donors. Ctrl: control; siNC: negative-control siRNA; siYAP1: YAP1-targeting siRNA. ** *p* < 0.01.

Ctrl + siYAP1-2 reduced GAG release into the medium at day 7 and day 21 compared to Ctrl + siNC, with the reduction at day 21 not reaching significance, whereas TGF-β1 + siYAP1-2 enhanced GAG release at day 21 but not at day 7 relative to TGF-β1 + siNC (Figure 6A,B). Furthermore, TGF-β1 + siYAP1-2 exhibited a non-significant trend toward increased COL2A1 mRNA expression at both time points, and a similar trend was observed for ACAN mRNA at day 7 but not at day 21 compared to TGF-β1 + siNC (Figure 6C–F). 

Notably, TGF-β1 + siNC decreased the GAG/DNA ratio at both day 7 and day 21 compared to TGF-β1 alone, although the difference was not significant at day 21 (Figure S1J,K). TGF-β1 + siNC elevated GAG release at day 7, but not day 21 compared to TGF-β1 alone (Figure 6A,B). To exclude potential siNC effects, we compared TGF-β1 + Mock (Lipofectamine™ RNAiMAX only) and TGF-β1 + siNC groups. In MSCs, YAP1 mRNA, YAP1 nuclear intensity, GAG/DNA, and GAG release were assessed at day 7 (Figure S2O–R). At day 7, TGF-β1 + siNC reduced GAG/DNA compared with TGF-β1 + Mock (Figure S2Q). Hence, the increase in GAG/DNA after YAP1 knockdown relative to siNC may not represent a direct YAP1 effect, and we therefore consider GAG/DNA an unreliable parameter in this context.

### 2.7. Effects of YAP1 Knockdown on Chondrogenic Matrix Deposition and Hypertrophic Marker Expression in MSC Pellets

**Figure 7:**
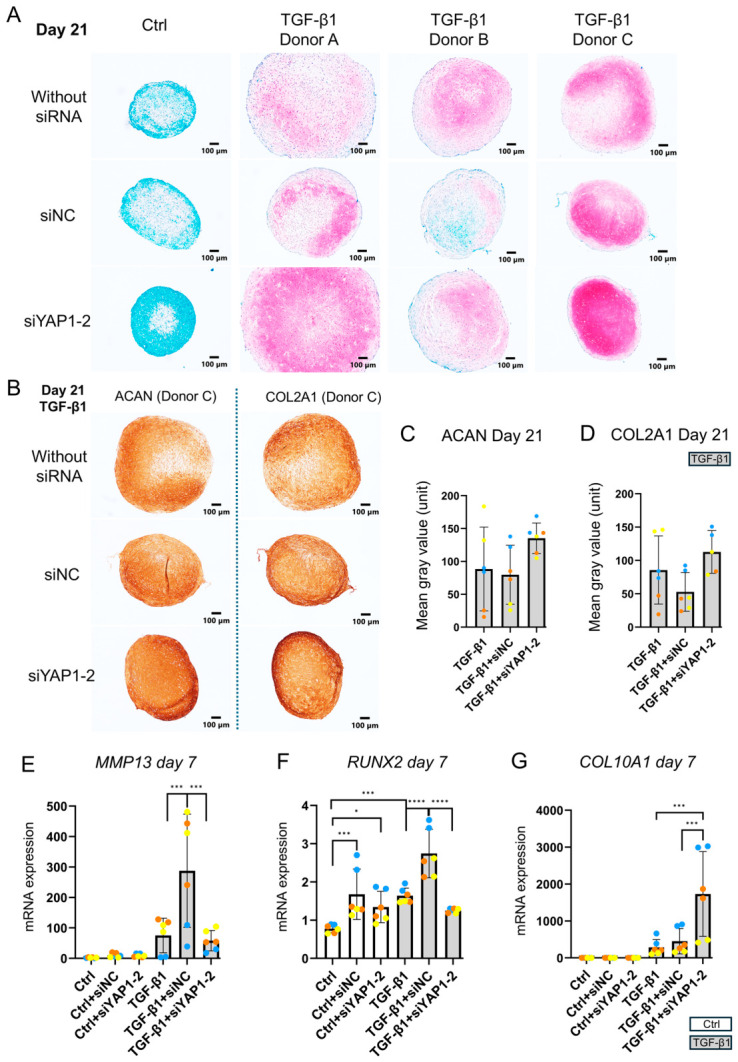
YAP1 knockdown enhances matrix deposition and alters hypertrophic markers. (**A**) Safranin O–Fast Green (SO-FG) staining of MSC pellets on day 21 following TGF-β1 stimulation and siRNA transfection. (**B**) Immunohistochemistry for ACAN and COL2A1 on day 21 with or without YAP1 knockdown. (**C**,**D**) Quantification of ACAN and COL2A1 staining intensity. (**E**–**G**) mRNA expression of MMP13, RUNX2, and COL10A1 at day 7 under indicated conditions. Different colors indicate individual donors. Ctrl: control; siNC: negative-control siRNA; siYAP1: YAP1-targeting siRNA. * *p* < 0.05, *** *p* < 0.001, **** *p* < 0.0001.

Compared to the control group, TGF-β1 treatment increased GAG staining in all three donors at day 21, as shown by Safranin O–Fast Green (SO-FG) staining (Figure 7A). Donors A and C exhibited higher staining in the TGF-β1 + siNC group, while Donor B showed reduced staining compared to TGF-β1 without siRNA. Notably, TGF-β1 + siYAP1-2 consistently enhanced GAG staining compared to TGF-β1 + siNC. siYAP1-2 also showed a trend toward increased ACAN and COL2A1 staining at day 21, though it was not statistically significant (Figure 7B–D). No observable changes in SO-FG, ACAN, or COL2A1 staining were found on day 7 (Supplementary Figure S3A–D).

Hypertrophic marker analysis revealed that in TGF-β1-treated pellets, siNC elevated MMP13 mRNA expression at day 7, whereas siYAP1-2 abolished the siNC induced increase (Figure 7E). Similarly, RUNX2 expression was upregulated by siNC in the presence of TGF-β1 but this was abolished by transfection with siYAP1-2 (Figure 7F). To exclude potential siNC effects, we compared the TGF-β1 + Mock (transfection reagent only) and TGF-β1 + siNC groups in MSCs at day 7. No significant differences were observed in the mRNA expression of MMP13 or RUNX2 (Figure S2S,T).

In contrast, COL10A1 expression was increased in the siYAP1-2 + TGF-β1 group compared to siNC + TGF-β1. No significant differences in hypertrophy markers were observed on day 21 (Supplementary Figure S3E–G).

**Figure 8:**
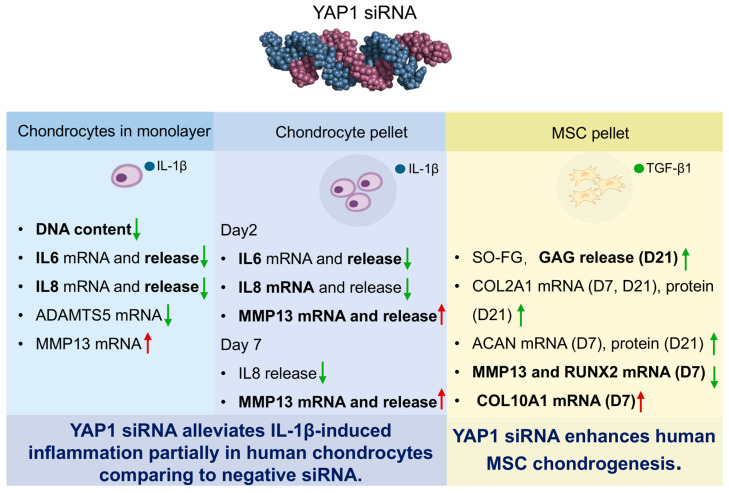
Summary of YAP1 knockdown effects on IL-1β-induced chondrocyte inflammation and TGF-β1-induced MSC chondrogenesis relative to negative siRNA. Statistical significance is indicated in bold; non-bold values represent consistent trends across all three donors without reaching significance. Upward arrows indicate upregulation and downward arrows indicate downregulation.

A summary of the overall effects of YAP1 knockdown is presented in Figure 8.

## 3. Discussion

Our study demonstrates that YAP1-targeting siRNA 2 (siYAP1-2) reduces the expression of IL-1β-induced pro-inflammatory mediators (IL-6, IL-8) in human chondrocytes in both monolayer and pellet cultures. Additionally, in TGF-β1-induced MSC pellets, siYAP1-2 enhanced COL2A1 and ACAN matrix deposition at day 21 and reduced RUNX2 and MMP13 mRNA levels at day 7, highlighting YAP1’s dual role in cartilage inflammation and regeneration (Figure 8). Some comparisons showed consistent trends that did not reach significance. Given the inherent inter-donor heterogeneity of primary chondrocytes [21] and MSCs [22], this cross-donor concordance is notable and suggests genuine biological effects that larger cohorts might resolve statistically.

In human chondrocyte monolayers, IL-1β (10 ng/mL) downregulated YAP1 mRNA expression at day 1 but not at day 3, whereas nuclear YAP1 levels were elevated at both time points. This suggests that IL-1β modulates YAP1 activity primarily through post-transcriptional mechanisms, promoting nuclear translocation independent of transcriptional regulation. In 3D chondrocyte pellets, IL-1β had no significant effect on YAP1 mRNA at days 2 or 7, though a non-significant increase in nuclear YAP1 was observed, further supporting regulation at the protein level.

IL-1β also promoted chondrocyte proliferation in monolayer cultures without FBS, as indicated by increased DNA content. However, one study has reported that IL-1β (10 ng/mL) reduced the proliferation of human chondrocyte cultured with 0.5% or 10% FBS [23]. This discrepancy may be due to the presence of serum, which contains growth factors and cytokines that can modulate IL-1β signaling [24] and alter proliferative responses.

In chondrocyte monolayer cultures, siYAP1-2 significantly downregulated YAP1 mRNA and reduced nuclear YAP1 intensity. In chondrocyte pellet cultures under IL-1β stimulation, siYAP1-2 significantly downregulated YAP1 mRNA at both day 2 and day 7. A corresponding significant reduction in nuclear YAP1 intensity was observed at day 7, with a non-significant reducing trend at day 2. Collectively, these results demonstrate the efficacy of siRNA-mediated YAP1 knockdown across distinct culture models.

YAP1 silencing attenuated IL-1β-induced proliferation, suggesting that YAP1 activity contributes to this response. Supporting this, previous studies have shown that YAP1 overexpression promotes ATDC5 cell proliferation, while YAP1 knockdown inhibits it [25]. Additionally, YAP1 has been reported to enhance chondrocyte proliferation through TEAD-mediated regulation of Sox6 [26]. However, conflicting evidence exists, as another study reported that YAP1 overexpression inhibited ATDC5 proliferation, and the knockdown of YAP1 was able to reverse the inhibition [27]. Notably, in 3D pellet cultures, IL-1β and siYAP1-2 had no effect on proliferation, likely due to the in-vivo-like properties of the 3D environment. This aligns with reports that YAP1/TAZ knockout impairs proliferation in vitro but not in vivo [28], emphasizing the influence of culture conditions on YAP1 function. Among the most important mechanical pathways influencing YAP are actomyosin contractility, substrate stiffness, cell–cell adhesion and nuclear mechanics [29]. These mechanical parameters differ fundamentally between 2D monolayer and 3D pellet cultures; notably, the rigid 2D substrate constitutively promotes YAP nuclear localization, whereas the softer, more physiologically compliant 3D environment attenuates this effect [11].

Knockdown of YAP1 partially attenuated IL-1β-induced inflammatory gene expression in human chondrocytes, as shown by reduced IL6 and IL8 levels in both monolayer and pellet cultures. YAP1 silencing also suppressed ADAMTS5 expression in IL-1β-treated 2D monolayers; however, no such effect was observed in 3D pellet cultures, likely because the pellet environment diminishes its influence on gene regulation. Despite these anti-inflammatory changes, YAP1 knockdown led to increased MMP13 expression. This paradoxical pattern of reduced IL6 and IL8 but persistent MMP13 upon YAP1 knockdown mirrors the conflicting literature on YAP1–NF-κB crosstalk. YAP1 has been reported both as an NF-κB co-activator that promotes inflammation [30] and as an NF-κB suppressor through TAK1-mediated inhibition [31]. In our study, YAP1 silencing attenuated IL-6 and IL-8, consistent with its co-activator function; conversely, the sustained MMP13 expression may reflect the loss of YAP1-mediated NF-κB suppression, consistent with its inhibitory role. This interpretation, however, remains speculative, as the precise mode of YAP1–NF-κB interaction likely depends on cellular context, promoter architecture, and disease stage. It is possible that YAP1 may differentially regulate inflammatory and matrix–remodeling pathways. Directly dissecting how YAP1 differentially regulates NF-κB target genes in human chondrocytes represents an important direction for future investigation. Beyond NF-κB, additional pathways may also contribute, such as YAP-dependent NLRP3 activation under mechanical stress [32]. Whether these or other mechanisms mediate the anti-inflammatory effects of siYAP1-2 awaits direct experimental investigation.

However, existing literature reflects contrasting roles of YAP1 in osteoarthritis (OA), with outcomes varying by species, IL-1β concentration, and OA induction model. In murine anterior cruciate ligament transection (ACLT)-induced OA, YAP1 expression in articular cartilage decreased with disease severity, and loss of YAP1 exaggerated cartilage destruction [31]. Similarly, YAP1 activation in destabilization of the medial meniscus (DMM)-induced mouse models attenuated OA progression by reducing chondrocyte apoptosis and cartilage breakdown [33]. In bovine chondrocytes, IL1β (1 ng/mL) reduced YAP1 activation, while mechanical stimulation or YAP1 agonists such as lysophosphatidic acid (LPA) mitigate IL-1β-induced inflammatory signaling [11]. These studies agree that the activation of YAP1 is beneficial to reduce OA progression in mice and bovine.

Conversely, other studies have reported an increased YAP1 expression in OA cartilage. In mice, IL-1β (10 ng/mL) upregulates YAP1 mRNA in chondrocytes, and YAP1 knockdown inhibited IL-1β-stimulated catabolic gene expression [12]. In rats, DMM surgery increased YAP1-positive chondrocytes, and siYAP1 reversed IL-1β (5 ng/mL)-induced ECM degradation and inflammation, whereas YAP1 overexpression worsened cartilage degeneration [30]. Additionally, microtubule stabilization promoted cartilage regeneration in rats by suppressing YAP1 activity [13]. These studies consistently show that YAP1 inhibition mitigates OA progression in murine and rat models.

To date, only one study has evaluated YAP1 in healthy human chondrocytes, reporting that increased nuclear YAP1 activation enhances ACAN, COL2A1, and SOX9 mRNA expression, while reduced nuclear YAP1 impairs anabolic activity [34]. Collectively, these findings emphasize that YAP1’s role in OA is highly context-dependent, influenced by intervention time, species, experimental model, cytokine concentration, and method of YAP1 modulation. Notably, our study is the first to demonstrate that siYAP1 reduces inflammatory responses in IL-1β-stimulated primary human chondrocytes.

We also observed that Lipofectamine™ RNAiMAX combined with siNC altered YAP1 expression and inflammatory responses in chondrocytes in a culture-dependent manner. In IL-1β-treated pellets, siNC reduced YAP1 mRNA, nuclear YAP1, and IL6, IL8, and MMP13 expression, particularly at day 2. These effects were absent at day 7; however, in monolayer cultures, IL-1β + siNC increased IL-6 and IL-8 secretion and protein/DNA ratios. Consistent with this, RNAiMAX with siNC did not induce inflammation of primary chondrocytes based on the expression of COX-2 in the context without IL-1β [35], supporting our observation that siNC-mediated inflammatory changes occurred only under IL-1β stimulation. Another study showed that siRNA–Lipofectamine 2000 complexes induced IL-8 in HeLa cells, whereas no induction was observed in PC3 or HEK293 cells, highlighting the cell-type-specific nature of this inflammatory response [36]. siRNA might be sensed by innate immune endosomal toll-like and cytoplasmic receptors, whose signaling elicits nonspecific effects, including inflammatory cytokines to trigger inflammation [37]. These findings highlight the influence of culture configuration on cellular responses and suggest that transfection itself may confound RNAi-based analyses of YAP1 and inflammation.

In TGF-β1-induced MSC chondrogenesis, YAP1 mRNA was downregulated while nuclear YAP1 protein increased by day 21, suggesting post-transcriptional regulation or nuclear translocation despite reduced transcripts. This is consistent with previous studies reporting decreased YAP1 mRNA expression during chondrogenic differentiation of mice [38]. Although reduced YAP1 mRNA levels were also observed in human MSCs from multiple tissues (synovial, periosteal, bone marrow) at early time points (days 1 and 6) [14], we did not observe such downregulation at day 7. The discrepancy may be due to differing chondrogenic protocols; the referenced study used micromass culture by seeding 4 × 10^5^ cells in 20 μL droplets [14], followed by serum-free medium after a 3 h attachment period without medium [39]. High YAP1 expression inhibits chondrogenic differentiation of murine C3H10T1/2 MSCs at day 7 [14], while YAP1 knockdown promotes chondrogenesis of human synovial mesenchymal stem cells at day 7 [40]. Collectively, our findings align with these observations and support the conclusion that YAP1 exerts inhibitory effects on chondrogenic differentiation. High YAP activity might impair chondrogenesis partly by suppressing Smad signaling and reducing BMP target genes *Id1*, *Id2*, and *Id3*; notably, *Id2* is required for BMP-induced chondrogenesis [14], yet whether other pathways contribute to this process remains to be determined.

Although YAP1 inhibition via siYAP1-2 improved matrix deposition on day 21 and suppressed RUNX2 and MMP13 on day 7, it upregulated COL10A1 expression, suggesting only partial attenuation of hypertrophy. MSC-derived cartilage often lacks the phenotype and mechanical integrity of native hyaline cartilage due to undesired hypertrophy, which must be minimized for clinical translation [41]. Our findings suggest that YAP1 inhibition may partially alleviate hypertrophic differentiation, but further optimization is required.

Finally, we found that siNC transfection upregulated YAP1 mRNA in the control groups at day 7 and day 21, and in TGF-β1 groups at day 7, without altering nuclear YAP1 levels. TGF-β1 + siNC also decreased GAG/DNA at days 7 and 21 compared to TGF-β1 alone, possibly due to increased YAP1 mRNA. Despite this, GAG release was elevated at day 7, suggesting impaired matrix retention rather than release. Additionally, TGF-β1 + siNC increased MMP13 and RUNX2 mRNA expression compared to TGF-β1 alone, indicating that transfection may exacerbate MSC hypertrophy and complicate interpretation of RNAi experiments targeting YAP1. Commercially available transfection components are not biologically inert [42], highlighting a critical consideration for the design and interpretation of RNAi experiments.

This study acknowledges several limitations. First, the sample size was small (*n* = 3), which limited statistical power and might explain why several biologically consistent trends did not reach statistical significance. Also, as the donor populations included only female chondrocyte donors and male MSC donors, sex-specific biological variability cannot be excluded. Moreover, the use of primary cells from distinct donors highlights donor variability, an intrinsic biological factor that can influence gene expression responses. Second, transfection-associated effects constitute a major technical limitation. Commercial transfection reagents are not biologically inert and can themselves alter inflammatory responses and chondrogenesis. Third, potential off-target effects of siRNA encompass both hybridization-dependent silencing of partially complementary transcripts [43] and hybridization-independent innate immune activation [44]. Fourth, the proposed mechanistic interpretations are literature-based and remain to be experimentally validated both in vitro and in vivo. There is an absence of protein-level validation by immunoblotting and a lack of rescue experiments to confirm siRNA specificity. Furthermore, confirmation in animal models of osteoarthritis is necessary to substantiate these hypotheses and strengthen the translational conclusions. Fifth, a technical limitation is that siRNA was delivered in monolayer prior to pellet formation, because direct transfection of pellets failed due to high cell density. Sixth, post-transfection time points may partially reflect transient, procedure-related perturbations rather than stable biological responses, indicating that longer culture periods and improved delivery strategies would strengthen the interpretation of mechanistic effects. Finally, although cartilage samples appeared macroscopically intact, their origin from osteoarthritic joints means that subclinical degenerative changes cannot be excluded; access to truly healthy cartilage would improve the generalizability of the findings.

## 4. Materials and Methods

### 4.1. Materials and Reagents

Dulbecco’s Modified Eagle’s Medium (DMEM) high glucose (Cat# 12100061), α Minimum Essential Medium (MEM, Cat# 61100087), penicillin-streptomycin (Pen/Strep, Cat# 15140122), Opti-MEM™ (Cat# 31985070), HEPES (Cat# 15630080), L-Glutamine (Cat# 25030081), and non-essential amino acids (Cat# 11140050) were purchased from Gibco (Waltham, MA, USA). ITS supplement (Cat# 354352) and fetal bovine serum (FBS) (Cat# 35-010-CV) were supplied by Corning (Corning, NY, USA). Human Animal-Free IL-1β protein (Cat# AF-200-01B) was purchased from Peprotech (Cranbury, NJ, USA). Recombinant human basic fibroblast growth factor (FGF-b, Cat# 30R-AF015) and recombinant human TGF-β1 protein (Cat# 30R-AT072) were supplied by Fitzgerald (New York, NY, USA). Collagenase II (Cat# LS004177) was purchased from Worthington Biochemical Corporation (Lakewood, NJ, USA). Lipofectamine™ RNAiMAX Transfection Reagent (Cat# 13778030) and TaqMan™ Reverse Transcription Reagents (Cat# 4370074) were purchased from Invitrogen (Carlsbad, CA, USA). Human IL6 (Cat# DY206), IL8 (Cat# DY208), total MMP13 (Cat# DY511), and ADAMTS5 ELISA kits (Cat# DY2198-05) were supplied by R&D Systems (Minneapolis, MN, USA). Cell culture chamber slide (8 wells) was purchased from SPL (The Woodlands, TX, USA) (Cat# 30108). Biotinylated Anti-Mouse IgG [H+L] made in horse (Cat# BA-2000-1.5), ABC-complex (Cat# PK-4000), and 3,3′-diaminobenzidine (DAB, Cat# SK-4105) were supplied by Vector Laboratories (Newark, CA, USA). Quant-iT PicoGreen dsDNA Assay Kits (Cat# P7589) were purchased from Invitrogen (Carlsbad, CA, USA). TRI reagent (Cat# TR 118) and PolyAcryl carrier (Cat# PC 152) were supplied by Molecular Research Center (Cincinnati, OH, USA). RNeasy Mini Kit (Cat# 74106) was purchased from Qiagen (Venlo, The Netherlands). TaqMan Gene Expression Master Mix (Cat# 4331182) was supplied by Applied Biosystems (Waltham, MA, USA). All other materials and reagents were purchased from Sigma-Aldrich (St. Louis, MO, USA).

### 4.2. Isolation and Expansion of Chondrocytes and MSCs

Cartilage fragments were dissected from the femoral head of patients (female; 58, 69, 73, or 78 years old), undergoing hip replacement operations with donors’ informed consent and ethical approval by cantonal ethical commission (KEK-ZH-NR: 2010-0444/0). Macroscopically intact cartilage tissue was minced into small pieces and digested in spinner flasks containing DMEM high glucose, 100 U/mL Pen/Strep, 10% FBS, and 450 U/mL collagenase II overnight at 37 °C and 5% CO_2_. Dissociated cells were cultured in expansion medium, which consisted of DMEM high glucose, 1% P/S, 10% FBS, 1 ng/mL TGF-β1, 5 ng/mL FGF-b, 10 mM HEPES, and 2 mM L-Glutamine. At 80% confluency, chondrocytes of passage 0 were detached and preserved in liquid nitrogen. After thawing and expanding, passage-three chondrocytes were used in this study. After expansion, chondrocytes were seeded at a low density [45] of 5 × 10^3^ cells/cm^2^ into distinct culture systems: an 8-well cell culture chamber slide for confocal imaging of YAP1 immunofluorescence, a 6-well plate for monolayer analysis, and a T300 flask for chondrocyte pellet studies. Cells were maintained in chondro-permissive medium consisting of high-glucose DMEM supplemented with 1% Pen/Strep, 50 µg/mL ascorbic acid, 100 nM dexamethasone, 1% ITS supplement, and 1% non-essential amino acids. This TGF-β-free medium maintained chondrocyte viability and phenotype [46]. The medium was changed every two days, and passage three was used for all experiments.

Human bone marrow was obtained from 3 donors (male; 48, 54 or 66 years old) with ethical approval from the local authorities (Freiburg, EK-326/08) and written consent of the patients undergoing total hip replacement. MSCs were isolated via density centrifugation separation and plastic adhesion and culture-expanded in αMEM supplemented with 10% FBS, 1% Pen/Strep and 5 ng/mL FGF-b at 37 °C, 5% CO_2_, and 95% humidity [47]. MSCs were seeded in T300 flasks at a density of 1 million/flask. The medium was changed every two days, and passage four was used for all experiments.

Donor information is provided in Table 1. All experiments were performed with cells from three independent donors, each assayed in duplicate. For nuclear YAP1 intensity quantification in monolayer chondrocytes, three randomly selected fields were imaged and analyzed per well.

### 4.3. siRNA Transfection

At around 70% confluency, cells were transfected using Lipofectamine™ RNAiMAX. Specifically, in a 6-well plate, 30 pmol siRNA was diluted in 150 μL of Opti-MEM™, and in a separate tube, 9 μL of Lipofectamine™ RNAiMAX was diluted in 150 μL of Opti-MEM™. The solutions were combined and gently mixed by pipetting, then incubated at room temperature for 5 min to allow complex formation. Finally, 300 μL of the mixture was added dropwise to each well of the 6-well plate. Cells were cultured for 2 days at 37 °C. Then, transfected cells were analyzed. Silencer™ Select Negative Control No. 1 siRNA (Invitrogen) was utilized as the negative control. YAP1 siRNAs were obtained from Life Technologies: siYAP1-1 (Catalog #4390818, Assay ID s20367) and siYAP1-2 (Catalog #4390818, Assay ID s534572). The sequences were as follows: siYAP1-1: UGAUUUAAGAAGUAUCUCUGA; siYAP1-2: UGUGAUUUAAGAAGUCUCT.

### 4.4. Pellet Culture of Chondrocytes and MSCs

After transfection in monolayer, chondrocytes were seeded at 0.25 × 10^6^ cells per well and MSCs at 0.2 × 10^6^ cells per well in V-bottom 96-well microplates. The plates were centrifuged at 400 × g for 5 min to form cell pellets. Chondrocyte pellets were cultured in 200 µL of chondro-permissive medium (DMEM high glucose, 1% Pen/Strep, 50 µg/mL ascorbic acid, 100 nM dexamethasone, 1% ITS supplement, 1% non-essential amino acids). To induce inflammation, 10 ng/mL IL-1β was added. MSC pellets were cultured in either chondro-permissive medium or chondrogenic medium supplemented with 10 ng/mL TGF-β1. Medium was refreshed and collected every two days. Chondrocyte pellets were harvested for analysis on days 2 and 7, whereas MSC pellets were harvested on days 7 and 21.

### 4.5. GAG and DNA Measurement

Each pellet was digested overnight at 56 °C in 500 µL proteinase K at 0.5 mg/mL. The total sulfated GAG contents in the pellet and in the medium were measured using the 1,9-dimethyl-methylene blue (DMMB) colorimetric method [48]. Briefly, 20 µL of digested pellet samples or 50 µL of conditioned medium were added into each well followed by 200 µL DMMB reagent. Absorbance at 535 nm was immediately read using a Tecan microplate reader (Tecan, Zürich, Switzerland). The proteinase K-digested samples were also used for measuring DNA content by the PicoGreen reagent, following the manufacturer’s instructions. Briefly, 100 µL of each sample was added into a 96-well white plate. An amount of 100 µL PicoGreen working solution was added and the fluorescence of the samples was measured at an excitation wavelength of 485 nm and emission of 535 nm by using a Tecan reader after incubation for 3 min at room temperature.

### 4.6. Safranin O/Fast Green Staining

Pellets were fixed in 10% buffered formalin overnight and dehydrated in an ascending ethanol series. Then, the samples were embedded in paraffin and sectioned at 6 µm thickness. To visualize the nuclei, slides were first stained with Weigert’s iron Haematoxylin for 10 min. After that, sections were stained with 0.02% Fast Green in ddH_2_O for 6 min, then stained with 0.1% Safranin O for 12 min to show proteoglycan deposition, and finally rinsed in ddH_2_O and sequentially dehydrated in graded ethanol. Finally, sections were mounted with Eukitt (Sigma-Aldrich, St. Louis, MO, USA). Images were acquired on an Olympus BX63 microscope (Hachioji, Tokyo, Japan).

### 4.7. Immunofluorescence of YAP1

Cells in monolayer were fixed with 4% formaldehyde for 30 min at room temperature, followed by two washes with PBS (5 min each). Permeabilization was performed with 0.1% TBST for 5 min, and blocking was carried out with 5% BSA in PBST for 1 h at room temperature. YAP1 primary antibodies, which are shown in Table 2, diluted in 1% BSA, were incubated overnight at 4 °C. After three 10 min washes with PBST, Alexa Fluor^®^ 488-conjugated anti-rabbit secondary antibody (1:500, Abcam ab150073) was applied for 1 h. Nuclei were stained with Hoechst 33342 (10 µg/mL) for 20 min. Following final washes, PBST was added, and samples were imaged by confocal microscopy. Samples stained with only secondary antibody were used as negative controls to assess nonspecific binding.

Paraffin sections were deparaffinized in xylene (2 × 5 min), rehydrated through graded ethanol (100%, 96%, 70%, 50%) to dH_2_O, and treated with 100% methanol for 2 min. Slides were washed in PBST (5 min) and blocked with 5% BSA in PBS for 1 h at room temperature. YAP1 antibodies were applied overnight at 4 °C. Following washes (3 × 10 min), Alexa Fluor^®^ 488-conjugated anti-rabbit secondary antibody was added for 1 h. After washing, slides were mounted with DAPI-containing medium and imaged by confocal microscopy. Images were analyzed and quantified using ImageJ software (Fiji distribution, version 2.14.0/1.54f). Samples incubated with secondary antibody alone served as negative controls to evaluate nonspecific binding.

### 4.8. Immunohistochemistry of ACAN and COL2A1

Paraffin sections were dewaxed and rehydrated in ethanol. Endogenous peroxidase activity was blocked by incubation with 0.3% hydrogen peroxide for 30 min. Then, pellet sections underwent different antigen retrieval protocols for the separate antibodies, which are shown in Table 2. Afterwards, non-specific antigen binding was blocked with horse serum for 1 h at room temperature. Samples were then incubated with primary antibody at 4 °C overnight. The sections were washed with PBST and then incubated with anti-mouse secondary antibody at room temperature for 30 min. After washing with PBST, the sections were incubated with ABC-complex at room temperature for 30 min. Following thorough washing with PBST, the sections were incubated with DAB for 4 min, followed by counterstaining with hematoxylin. The sections were then washed and dehydrated. Finally, sections were mounted with Eukitt. Images were acquired on an Olympus BX63 microscope. Images were analyzed and quantified using ImageJ software.

### 4.9. Interleukin-6, Interleukin-8, Matrix Metallopeptidase 13, and ADAMTS5 Release

Human interleukin-6 (IL 6), interleukin-8 (IL 8), Matrix Metallopeptidase 13 (MMP13), and A Disintegrin and Metalloproteinase with Thrombospondin Motifs 5 (ADAMTS5) released by chondrocytes were measured by using a commercial enzyme-linked immunosorbent assay (ELISA) kit according to manufacturers’ instructions. Cytokine amounts were normalized to total DNA content when experimental conditions altered DNA quantification; otherwise, raw cytokine amounts were compared directly.

### 4.10. Gene Expression Analysis

RNA was extracted from a pool of two pellets with 1 mL TRI Reagent and 5 µL PolyAcryl carrier, then homogenized with a Retsch tissue lyser (Retsch, Haan, Germany) at 30 Hz for 6 min. Monolayer chondrocytes were not subjected to homogenization. After centrifugation, the supernatant was collected. A total of 0.1 mL of 1-Bromo-3-chloropropane (BCP) was added to get the aqueous phase containing RNA, which was purified with an RNeasy Mini Kit. Reverse transcription was conducted with TaqMan™ Reverse Transcription Reagents. qRT-PCR was performed with TaqMan gene expression master mix on a QuantStudio™ 6 Pro Real-Time PCR system (Thermo Fisher, Waltham, MA, USA). Primer and probes sequences or assay-on-demand numbers are presented in Table 3. Relative quantification of mRNA was performed according to the 2^−∆∆Ct^ method with RPLP0 as the endogenous control. Gene expression values were normalized to the average values on day 0.

### 4.11. Statistical Analysis

The data are presented as the mean ± standard deviation (SD) of three independent donors. Shapiro–Wilk normality test was used to define whether the data were normally distributed in GraphPad Prism 8.1.0 software. An analysis of variance (ANOVA) was conducted to determine differences with normal distribution. Multiple comparison was corrected by the Tukey test following the ANOVA. The Friedman test was conducted to evaluate the differences for non-normally distributed data. Multiple comparison was corrected by Dunn’s test following the Friedman test. A *p* value < 0.05 was considered significant.

## 5. Conclusions

YAP1 knockdown reduces inflammation in human chondrocytes and enhances chondrogenesis while partially limiting hypertrophy in human MSCs. These findings suggest that YAP1 modulation may represent a potential therapeutic strategy for osteoarthritis by simultaneously reducing cartilage inflammation and enhancing cartilage regeneration capacity. However, its effects are context-dependent, and transfection may confound outcomes. YAP1 remains a complex target for OA therapy that should be further investigated. Future studies should validate these observations using alternative YAP1 modulation strategies and in preclinical models, with the aim of refining YAP1-based interventions for cartilage repair and regeneration.

## Figures and Tables

**Table 1:** Demographic characteristics of patients included.

Donors	Cell Type	Gender	Age (Years)	Kellgren–Lawrence Grade
a	Chondrocyte	Female	78	IV
b	Chondrocyte	Female	58	I
c	Chondrocyte	Female	73	Not available
d	Chondrocyte	Female	78	II
A	MSC	Male	54	-
B	MSC	Male	66	-
C	MSC	Male	48	-

**Table 2:** Antibodies used for immunofluorescence and immunohistochemistry staining.

Antibody	Company, Catalog	Digestion	Working Concentration
YAP1	Thermo Fisher, PA5-78321	Without digestion.	1/500
COL2A1	Thermo Fisher, MA5-12789	1 mg/mL pepsin in Tris-HCl, pH 2.0 for 10 min at 37 °C.	1/200
ACAN	DSHB, 12/21/1-C-6	0.25 U/mL Chondroitinase ABC and 2000 U/mL Hyaluronidase in PBST for 30 min at 37 °C.	4 µg/mL

**Table 3:** Primers and probes used for PCR.

Gene	Forward Primer	Reverse Primer	Probe (5′→3′)
*RPLP0*	5′-TGG GCA AGA ACA CCA TGA TG-3′	5′-CGG ATA TGA GGC AGC AGT TTC-3′	5′-AGG GCA CCT GGA AAA CAA CCC AGC-3′
*MMP13*	5′-CGG CCA CTC CTT AGG TCT TG-3′	5′-TTT TGC CGG TGT AGG TGT AGA TAG-3′	5′-CTC CAA GGA CCC TGG AGC ACT CAT GT-3′
*COL2A1*	5′-GGC AAT AGC AGG TTC ACG TAC A-3′	5′-GAT AAC AGT CTT GCC CCA CTT ACC-3′	5′-CCT GAA GGA TGG CTG CAC GAA ACA TAC-3′
*ACAN*	5′-AGT CCT CAA GCC TCC TGT ACT CA-3′	5′-CGG GAA GTG GCG GTA ACA-3′	5′-CCG GAA TGG AAA CGT GAA TCA GAA TCA ACT-3′
*RUNX2*	5′-AGC AAG GTT CAA CGA TCT GAG AT-3′	5′-TTT GTG AAG ACG GTT ATG GTC AA-3′	5′-TGA AAC TCT TGC CTC GTC CAC TCC G-3′
*COL10A1*	5′-ACG CTG AAC GAT ACC AAA TG-3′	5′-TGC TAT ACC TTT ACT CTT TAT GGT GTA-3′	5′-ACT ACC CAA CAC CAA GAC ACA GTT CTT CAT TCC-3′
*YAP1*	Hs00902712_g1		
*IL6*	Hs00174131_m1		
*IL8*	Hs00174103_m1		
*ADAMTS5*	Hs01095518_m1		

Primers and probes presented with sequences were custom designed; primers and probes presented with catalogue numbers were from Applied Biosystems.

## Data Availability

The original contributions presented in this study are included in the article/Supplementary Material. Further inquiries can be directed to the corresponding author.

## Supplementary Materials

The following supporting information can be downloaded at: https://www.mdpi.com/article/10.3390/ph19060859/s1, Figure S1: Effects of YAP1 knockdown on chondrocytes and MSCs. Figure S2: Analysis of YAP1 knockdown effects in chondrocyte and MSC pellet cultures. Figure S3: YAP1 knockdown did not affect matrix deposition of MSCs on day 7 or hypertrophic markers on day 21.

## Abbreviations

The following abbreviations are used in this manuscript:

ACAN	Aggrecan
ADAMTS5	A Disintegrin and Metalloproteinase with Thrombospondin Motifs 5
COL2A1	Collagen Type II Alpha 1 Chain
COL10A1	Collagen Type X Alpha 1 Chain
DAB	3,3′-Diaminobenzidine
DMMB	Dimethylmethylene Blue
ELISA	Enzyme-Linked Immunosorbent Assay
FGF-b	Fibroblast Growth Factor–Basic
IL-1β	Interleukin 1 Beta
IL6	Interleukin 6
IL8	Interleukin 8
ITS	Insulin–Transferrin–Selenium Supplement
MMP13	Matrix Metallopeptidase 13
MSC	Mesenchymal Stem Cell
OA	Osteoarthritis
qRT-PCR	Quantitative Reverse Transcription Polymerase Chain Reaction
RPLP0	Ribosomal Protein Lateral Stalk Subunit P0
RUNX2	Runt-Related Transcription Factor 2
siNC	Small Interfering RNA, Negative Control
siYAP1	Small Interfering RNA Targeting Yes-Associated Protein 1
sGAG	Sulfated Glycosaminoglycans
SO-FG	Safranin O–Fast Green
TGF-β1	Transforming Growth Factor Beta 1
TNF-α	Tumor Necrosis Factor Alpha
YAP1	Yes-Associated Protein 1
