# Supplementary material for: YAP1 Knockdown Reduces IL-1β-Induced Human Chondrocyte Inflammation and Promotes Human MSC Chondrogenesis
Source: Pharmaceuticals (Basel). 2026 May 29;19(6):859. doi: 10.3390/ph19060859 (PMC13304492; doi:10.3390/ph19060859)
Supplement: Supplementary file 1 [file pharmaceuticals-19-00859-s001.zip › pharmaceuticals-4298323-supplementary.pdf]

pharmaceuticals

MDPI

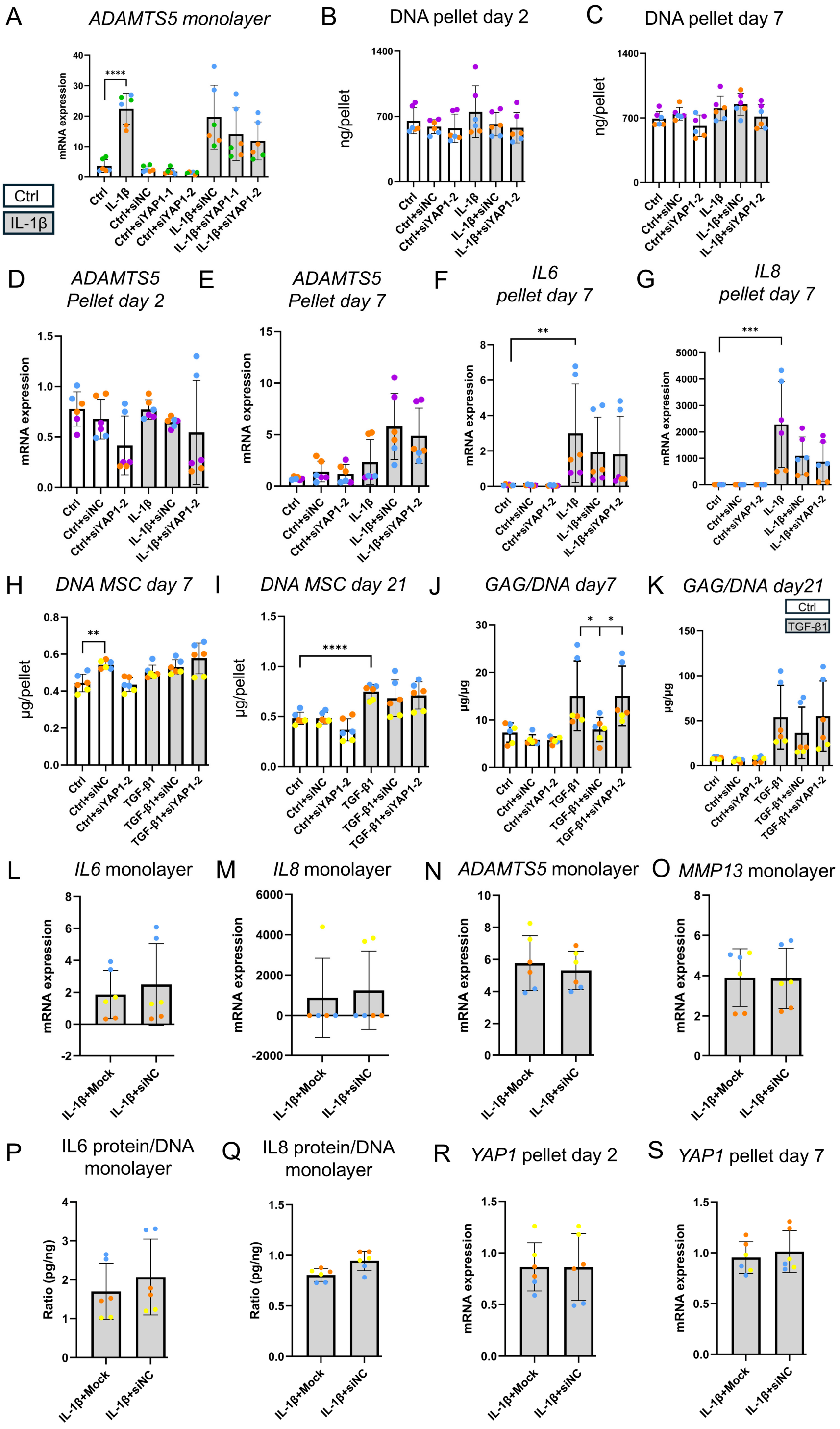

Figure S1. Effects of YAP1 knockdown on chondrocytes and MSCs. (A) mRNA expression of ADAMTS5 in chondrocyte monolayers at day 3. (B, C) DNA content in chondrocyte pellets with or without YAP1 knockdown at day 2 and day 7. (D, E) mRNA expression of ADAMTS5 in chondrocyte pellets at day 2 and day 7. (F, G) mRNA expression of IL6 and IL8 in chondrocyte pellets at day 7. (H, I) DNA content in MSC pellets at day 7 and day 21. (J, K) GAG/DNA in MSC pellets at day 7 and day 21. (L-O) mRNA expression of IL6, IL8, ADAMTS5, and MMP13 in chondrocyte monolayers at day 3. (P, Q) Levels of IL6 and IL8 normalized to DNA content in chondrocyte monolayers at day 3. (R, S) YAP1 mRNA expression nuclear intensity in chondrocyte pellets at day 2 and day 7.

Data are presented as paired samples from individual donors (indicated by colors). Comparisons between two groups were performed using paired t-tests for normally distributed data or Wilcoxon matched-pairs signed rank tests for non-parametric data. *P < 0.05, **P < 0.01. Ctrl, untreated; siNC, negative control siRNA; Mock, transfection reagent only.

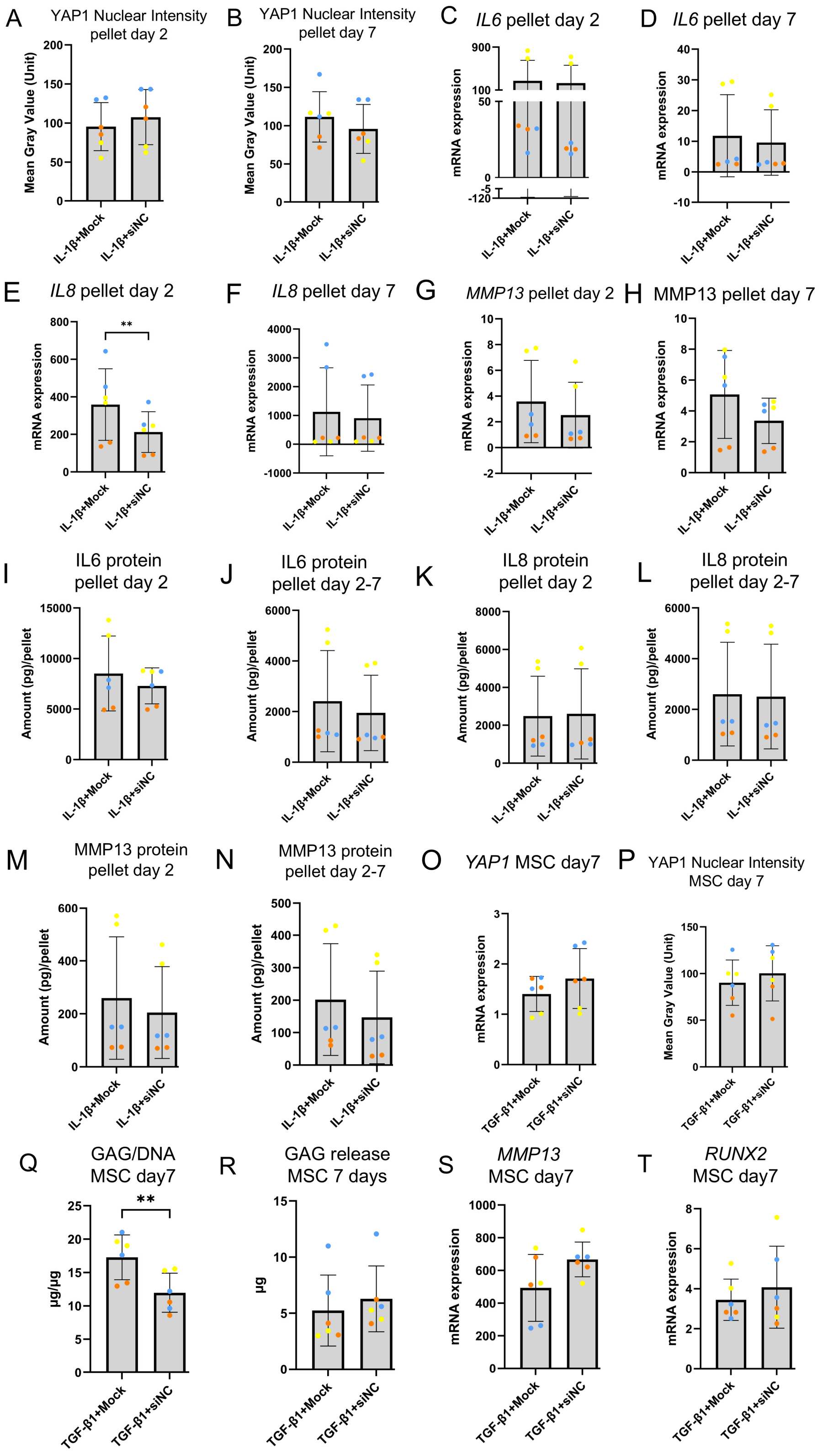

Figure S2. Analysis of YAP1 knockdown effects in chondrocyte and MSC pellet cultures. (A, B) YAP1 nuclear intensity in chondrocyte pellets at day 2 and day 7. (C-H) mRNA expression of IL6, IL8, and MMP13 in chondrocyte pellets at day 2 and day 7. (I-N) Protein levels of IL6, IL8, and

https://doi.org/10.3390/ph19060859

MMP13 in chondrocyte pellets at day 2 and from day 2 to day 7. (O, P) YAP1 mRNA expression and nuclear intensity in MSC pellets at day 7. (Q, R) GAG content normalized to DNA and GAG release in MSC pellets at day 7. (S, T) mRNA expression of MMP13 and RUNX2 in MSC pellets at day 7. Ctrl, untreated; siNC, negative control siRNA; Mock, transfection reagent only. **P < 0.01.

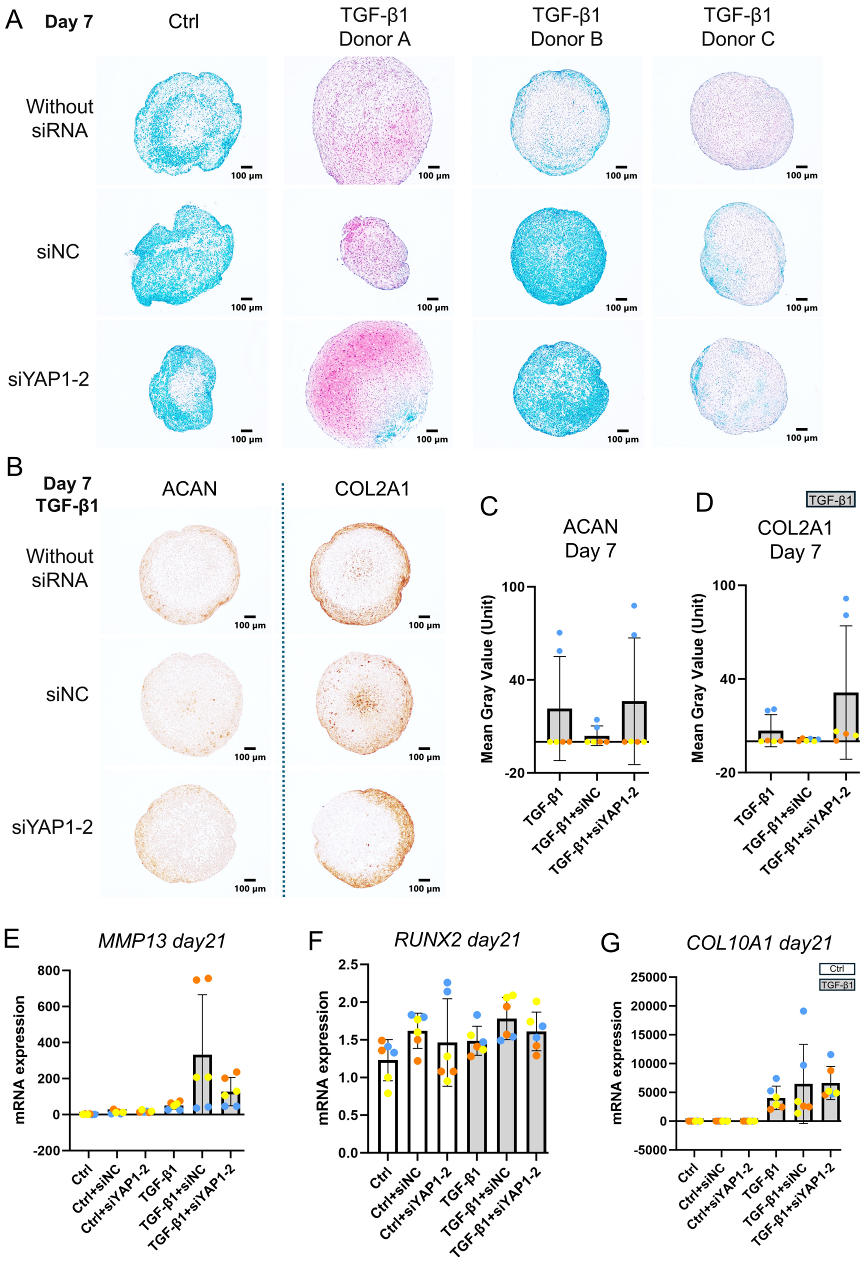

Figure S3. YAP1 knockdown did not affect matrix deposition of MSCs on day 7 or hypertrophic markers on day 21. (A) Safranin O–Fast Green (SO-FG) staining of MSC pellets on Day 7 following TGF-β1 stimulation and siRNA transfection. (B) Immunohistochemistry for ACAN and COL2A1 on Day 7 with or without YAP1 knockdown. (C, D) Quantification of ACAN and COL2A1 staining intensity. (E–G) mRNA expression of MMP13, RUNX2, and COL10A1 at Day 21 under indicated conditions.

https://doi.org/10.3390/ph19060859
